# The therapeutic potential of psychedelic drugs: past, present, and future

**DOI:** 10.1192/j.eurpsy.2026.10407

**Published:** 2026-07-31

**Authors:** D. Nutt

**Affiliations:** Psychiatry, Imperial College London, London, United Kingdom

## Abstract

**Abstract:**

The last decade has seen a remarkable resurgence of interest in psychedelic drugs such as psilocybin (from magic mushrooms) LSD and DMT (dimethyl tryptamine – the active ingredient of ayahuasca). This has been driven by the discovery that these psychedelics all act agonists of 5-HT2A receptors plus human imaging studies that reveal this action leads to profound alterations in brain measures of activity particularly in terms of increased entropy of EEG MEG and fMRI signals and reduced within-network, but increased between-network, connectivity. In addition they all can increase synaptic growth and brain plasticity. These findings not only explain the subjective nature of the psychedelic experience but also have implications for the treatment of internalising disorders such as depression addiction anorexia and OCD. Subsequent trials, particularly of psilocybin, in these disorders has revealed significant clinical benefits from even just a single administration. My talk will explore these brain mechanisms and clinical data and discuss the potential place of psychedelic medicine in the future of psychiatry.

**Disclosure of Interest:**

D. Nutt Shareolder of: chief scientific officer Solvonis Therapeutics ltd, Grant / Research support from: Support for psychedelic research from Compasspathways, Beckley Psytec, Usona, Filament, Small Pharma, Employee of: chief scientific officer Solvonis Therapeutics ltd.

